# A new genome-scale metabolic model of *Corynebacterium glutamicum* and its application

**DOI:** 10.1186/s13068-017-0856-3

**Published:** 2017-06-30

**Authors:** Yu Zhang, Jingyi Cai, Xiuling Shang, Bo Wang, Shuwen Liu, Xin Chai, Tianwei Tan, Yun Zhang, Tingyi Wen

**Affiliations:** 1 CAS Key Laboratory of Pathogenic Microbiology and Immunology, Institute of Microbiology, Chinese Academy of Sciences, Beijing, 100101 China; 2 University of Chinese Academy of Sciences, Beijing, 100049 China; 3 Beijing University of Chemical Technology, Beijing, 100029 China; 4 Savaid Medical School, University of Chinese Academy of Sciences, Beijing, 100049 China

**Keywords:** *Corynebacterium glutamicum*, Genome-scale metabolic model, l-proline production, Model-driven experimentation, Metabolic engineering

## Abstract

**Background:**

*Corynebacterium glutamicum* is an important platform organism for industrial biotechnology to produce amino acids, organic acids, bioplastic monomers, and biofuels. The metabolic flexibility, broad substrate spectrum, and fermentative robustness of *C. glutamicum* make this organism an ideal cell factory to manufacture desired products. With increases in gene function, transport system, and metabolic profile information under certain conditions, developing a comprehensive genome-scale metabolic model (GEM) of *C. glutamicum* ATCC13032 is desired to improve prediction accuracy, elucidate cellular metabolism, and guide metabolic engineering.

**Results:**

Here, we constructed a new GEM for ATCC13032, *i*CW773, consisting of 773 genes, 950 metabolites, and 1207 reactions. Compared to the previous model, *i*CW773 supplemented 496 gene–protein-reaction associations, refined five lumped reactions, balanced the mass and charge, and constrained the directionality of reactions. The simulated growth rates of *C. glutamicum* cultivated on seven different carbon sources using *i*CW773 were consistent with experimental values. Pearson’s correlation coefficient between the *i*CW773-simulated and experimental fluxes was 0.99, suggesting that *i*CW773 provided an accurate intracellular flux distribution of the wild-type strain growing on glucose. Furthermore, genetic interventions for overproducing l-lysine, 1,2-propanediol and isobutanol simulated using OptForce_MUST_ were in accordance with reported experimental results, indicating the practicability of *i*CW773 for the design of metabolic networks to overproduce desired products. In vivo genetic modifications of *i*CW773-predicted targets resulted in the de novo generation of an l-proline-overproducing strain. In fed-batch culture, the engineered *C. glutamicum* strain produced 66.43 g/L l-proline in 60 h with a yield of 0.26 g/g (l-proline/glucose) and a productivity of 1.11 g/L/h. To our knowledge, this is the highest titer and productivity reported for l-proline production using glucose as the carbon resource in a minimal medium.

**Conclusions:**

Our developed *i*CW773 serves as a high-quality platform for model-guided strain design to produce industrial bioproducts of interest. This new GEM will be a successful multidisciplinary tool and will make valuable contributions to metabolic engineering in academia and industry.

**Electronic supplementary material:**

The online version of this article (doi:10.1186/s13068-017-0856-3) contains supplementary material, which is available to authorized users.

## Background

*Corynebacterium glutamicum*, a Gram-positive soil bacterium, is an important platform organism for industrial biotechnology and environmental remediation [1, 2]. This microorganism is generally recognized as safe and is used in the fermentative production of l-glutamate and l-lysine on a million-ton scale and l-threonine, l-leucine and l-valine on a thousand-ton scale in the food, feed and pharmaceutical industries [1, 3]. Due to the metabolic flexibility, broad substrate spectrum, and fermentative robustness of *C. glutamicum* cells, this bacterium has attracted attention as a potential cell factory to manufacture organic acids, bioplastic monomers (e.g., putrescine and cadaverine), and biofuels including ethanol, isobutanol, 1,2-propanediol, and 1-propanol [1, 4, 5]. Based on the knowledge of biochemical pathways and microbial fermentation processes, new technologies such as systems metabolic engineering and high-throughput analyses at the genome, transcriptome, proteome, and fluxome levels have been applied to strain development to improve the yield and productivity of desired products [6–10]. In silico genome-wide predictive simulation of metabolic profiles is expected to enable the design of artificial metabolic networks for desired product synthesis [11].

A genome-scale metabolic model (GEM) is constructed based on experimental data and information on gene annotations and functions, metabolites, reactions, enzymes, and their interactions [11, 12]. GEM has become an essential tool for understanding cellular metabolism, characterizing cell phenotypes, designing mutant strains with desired properties, and assessing the effects of genetic intervention and environmental perturbation on cellular metabolism [13]. The quality of a GEM determines the accuracy of outcomes for describing the genotype–phenotype relationship of a given strain [13, 14]. The first GEM of *Escherichia coli* (*i*JE660) consisted of 660 genes, 627 reactions, and 438 metabolites. *i*JE660 was constantly updated to *i*JO1366 by increasing the numbers of new genes and reactions, expanding the gene-protein-reaction associations (GPRs), balancing the reactions and compartmentalizing metabolites, among other updates [15–18]. The updated GEM accurately characterized *E. coli* growth metabolic profiles and was used to perform computational and quantitative analyses to resolve problems [14]. When coupled with metabolic flux constraints and a cellular objective, GEM analysis using computational algorithms [e.g., flux balance analysis (FBA), OptKnock, or OptForce] can predict the modification of targets in a metabolic network and successfully improve the production of succinic acid, ethanol, and malonyl-CoA in *E. coli* [19–21].

The initial GEM of *C. glutamicum* ATCC13032 (Model_Cg_ 1) consisted of 247 genes, 446 reactions, and 411 metabolites and was updated to Model_Cg_ 2 (277 genes, 502 reactions, and 423 metabolites) by revising inadequate reaction loops [22, 23]. Although these two models are useful, they have several limitations due to previously inadequate information. Annotated genes known to participate in different metabolic pathways, such as pyruvate kinase isoenzyme, glycerol-3-phosphatase, and (S,S)-butanediol dehydrogenase, were not included in Model_Cg_ 1 or in Model_Cg_ 2 [24–26]. The deficiency of corresponding metabolic pathways in the two models prevented the prediction of cell growth on certain carbon sources (e.g., acetate, lactate, and xylose). The imbalance of the mass and charge in a few reactions (e.g., synthesis of RNA, protein, and biomass) and the directionality of certain reactions without thermodynamic constraints had a negative effect on genome-wide prediction using the two models. Moreover, the two models have incomplete l-glutamate metabolic systems, which prevented their application in studies of the metabolic state of l-glutamate production in the stationary phase [22, 27]. In 2016, Mei et al. constructed *i*JM658 based on the genome information of *C. glutamicum* S9114, which was generated by mutagenesis and is widely used for industrial l-glutamate production [27]. *i*JM658 contains l-glutamate secretion and uptake systems, which could be more suitable for studying l-glutamate production [27]. *i*JM658 is not applicable for the prediction of metabolic profile of *C. glutamicum* ATCC13032 due to the presence of numerous differences in the genome sequences between *C. glutamicum* S9114 and ATCC13032. Therefore, it is necessary to expand the scope of *C. glutamicum* ATCC13032’ GEM to metabolic engineering for the desired products.

In this study, we present an updated model of the *C. glutamicum* ATCC13032 metabolic network reconstruction. This new model, named *i*CW773, accurately predicted the growth ability and flux distributions of cells cultivated under different growth conditions. *i*CW773 simulations of genetic interventions for the overproduction of l-lysine, 1,2-propanediol, and isobutanol were highly consistent with experimental data. Along with wet-lab practices, genetic modifications were performed to generate a de novo l-proline-overproducing strain that exhibited the highest reported titer and productivity using a minimal medium in fed-batch fermentation.

## Results and discussion

### A new genome-scale metabolic model of *C. glutamicum* ATCC13032

We constructed a new GEM for ATCC13032, *i*CW773, which consisted of chemical reactions and interconverted metabolites. This network reconstruction contained 773 genes, 951 metabolites, and 1207 reactions, representing considerable increases in the numbers of genes, reactions and metabolites by 2.79-, 2.41-, and 2.25-fold, respectively, over Model_Cg_ 2 (Additional file 1: Table S1). In the internal reactions of *i*CW773, 709 ORFs (89.18%) were extracted from the KEGG and UniProt databases [28]. The total ORF coverage was 25.75% in *i*CW773, which was 16.52% higher than that in Model_Cg_ 2 (Additional file 1: Table S1). Five lumped reactions for fatty acids biosynthesis in Model_Cg_ 2 were refined in *i*CW773 by replacing each lumped reaction with several sequential reactions in *i*CW773 (Additional file 2). Additionally, the charge and mass of reactions in *i*CW773 were balanced, and the mass of reactions related to biomass was tested to be 1.00 g/g dry cell weight (DCW) (Additional file 3). The directionality of reactions was constrained by the Gibbs free energy Δ_*r*_ *G*′, which was calculated using eQuilibrator^2.0^ [29]. A complete list of all the reactions and metabolites for *i*CW773 is provided in Additional file 3, and the central carbon metabolic pathway is presented in Additional file 4: Figure S1.

### Prediction of growth rates under different culture conditions

**Fig. 1:**
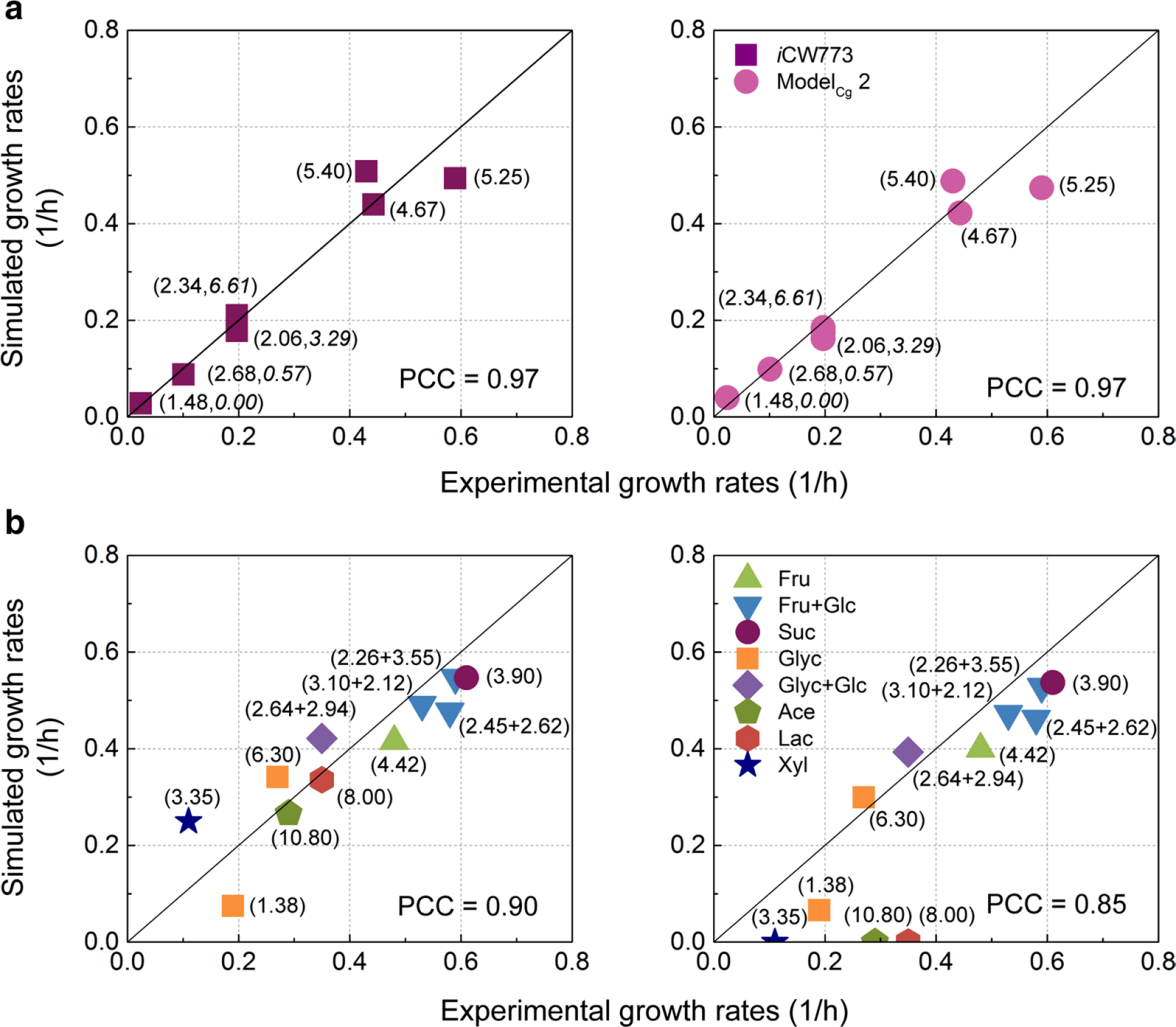
Comparison of growth rates between experimental data and in silico simulation results at various glucose and oxygen supply levels (**a**) and different carbon sources (**b**) using *i*CW773 (*left*) and Model_Cg_ 2 (*right*). PCC is the Pearson’s correlation coefficient between the predicted product yield values and experimental data. The line corresponding to *x* = *y* is also shown. **a** The glucose uptake rate is shown in *regular font* in *parentheses*, and the oxygen uptake rate is shown in *italics* in *parentheses*. **b** The uptake rate of the corresponding carbon source is shown in *parentheses*

To evaluate the quality of *i*CW773, the growth rates of *C. glutamicum* ATCC13032 cultivated on different carbon sources were predicted through FBA simulations using biomass maximization as the objective function. First, we calculated the growth rates on glucose using GEMs by setting the glucose and oxygen consumption rates as the experimental values [22, 30–32]. As shown in Fig. 1a, the Pearson’s correlation coefficient (PCC) between the experimental and *i*CW773-simulated data was consistent with that between the experimental and Model_Cg_ 2-simulated data. When fructose, fructose mixed with glucose, sucrose, acetate, and lactate were set as the carbon sources, the cell growth rates on acetate or lactate could be calculated only by *i*CW773, rather than Model_Cg_ 2 (Fig. 1b) [33–37]. It has been reported that *C. glutamicum* was able to grow on non-native glycerol or xylose as the sole carbon source after expressing heterologous-related enzymes [38–40]. When the three reported reactions catalyzed by glycerol kinase, glycerol 3-phosphate dehydrogenase, and aquaglyceroporin from *E. coli* were added, both GEMs could calculate the biomass from glycerol [38, 39]. In contrast, only *i*CW773 could simulate the biomass accumulation from xylose (Fig. 1b) when two reported reactions catalyzed by xylose isomerase and xylulokinase from *E. coli* were added [40]. These results provided evidence that supplemental reactions in *i*CW773 coupled the metabolism of the corresponding substrates (e.g., acetate, lactate, and xylose) with the central carbon metabolism (Additional file 5), resulting in the realization of growth simulations on these substrates.

### Metabolic profile prediction using *i*CW773

**Fig. 2:**
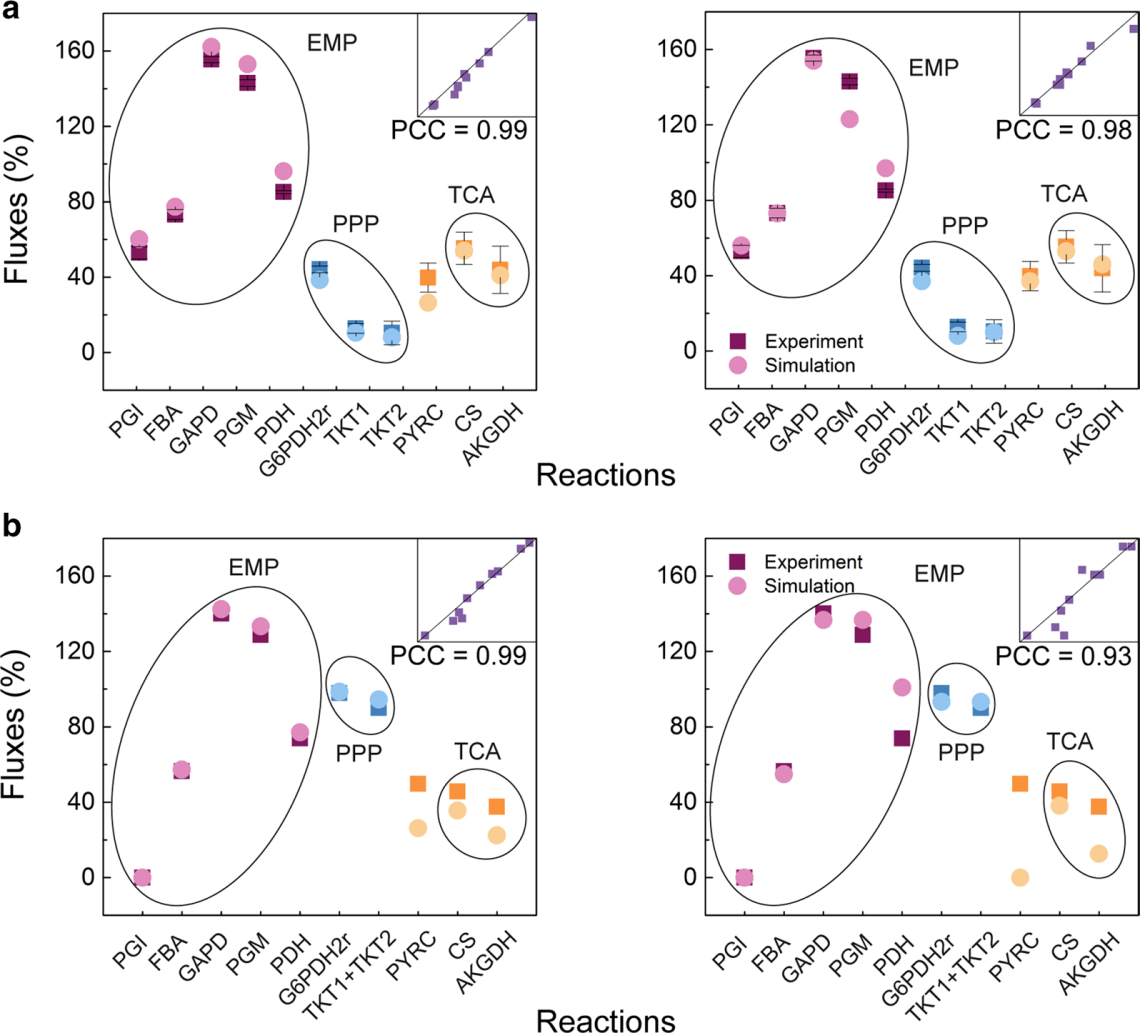
Comparison of the metabolic flux profiles of the central metabolism between the ^13^C-tracer experimental WT (**a**) and WTΔ*pgi* (**b**) data for the exponential growth phase and in silico simulation results using *i*CW773 (*left*) and Model_Cg_ 2 (*right*). Fluxes were normalized to the glucose uptake rates, which were set to 100. PCC is the Pearson’s correlation coefficient between the predicted product yield values and experimental data. The line corresponding to *x* = *y* is also shown in the inserted figure in the *top right corner*. EMP: Embden–Meyerhof pathway (glycolytic pathway); PPP: pentose phosphate pathway; TCA: tricarboxylic acid cycle. **a** The experimental data were derived from three studies, and the *error bars* represent the standard deviations [8, 31, 41]

Next, we compared model-predicted metabolic flux distributions to ^13^C-tracer metabolic flux data from ATCC13032 cultivated in minimal medium using glucose as the sole carbon source [8, 31, 41]. The biomass production was used as the objective function, and all flux data were normalized to the glucose uptake rate (100%). Notably, the PCC between the experimental and *i*CW773-simulated fluxes was 0.99, suggesting that *i*CW773 provided an accurate intracellular flux distribution of the WT strain growing on glucose (Fig. 2a).

It has been reported that the inactivation of *pgi*-encoding glucose-6-phosphate isomerase can lead to a significant metabolic flux disturbance, blocking the EMP pathway and redirecting the carbon flux from glucose-6-phosphate toward the PPP pathway in *C. glutamicum* [31]. To analyze the metabolic disturbance by gene knockout, the *pgi* gene was deleted in *i*CW773 and Model_Cg_ 2. When FBA was used to calculate the intracellular metabolic flux, the *i*CW773-predicted flux toward the PPP pathway increased from 38.49 to 98.59%, which was consistent with the experimental values (from 44.10 to 97.80%) (Fig. 2b) [31]. Moreover, the simulated amount of NADPH generated from the PPP pathway in WT∆*pgi* was 1.97 mol/mol glucose, which was 2.30-fold higher than that generated from the PPP pathway in WT (0.77 mol/mol glucose). Due to the requirement for a certain amount of NADPH to generate biomass in the simulation (e.g., 4 mol NADPH per mol l-lysine and 2 mol NADPH per mol l-valine), the NADPH generated from isocitrate dehydrogenase in the TCA cycle decreased from 0.41 mol/mol glucose to 0.22 mol/mol glucose to maintain a stoichiometric balance [42, 43]. This difference in NADPH generation resulted in a smaller deviation from the *i*CW773-simulated TCA flux to the experimental value, as shown in Fig. 2b (left). Notably, the flux of pyruvate carboxylase (PYRC) in *i*CW773 was efficiently generated, while no PYRC flux from pyruvate to oxaloacetate was calculated in Model_Cg_ 2. These results illustrate the suitability of *i*CW773 for predicting intracellular metabolic flux distribution.

### Validation of *i*CW773 simulation for amino acid overproduction

**Fig. 3:**
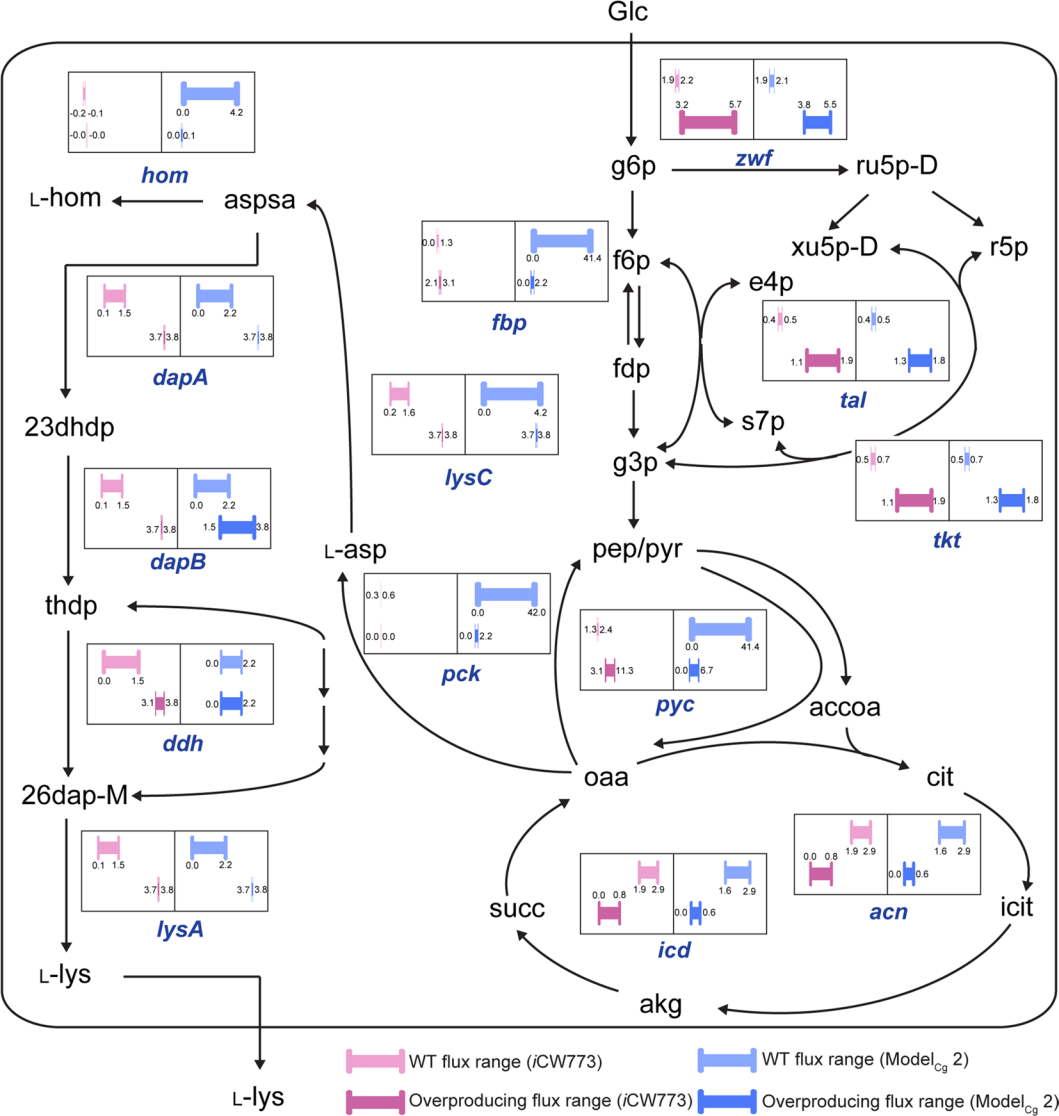
Application for overproduction of l-lysine using OptForce_MUST_. Comparison of the flux ranges for reactions in *i*CW773 and Model_Cg_ 2. Genes encoding relevant enzymes are depicted with *italics* next to the *arrows*: *acn*: aconitase; *dapA*: dihydrodipicolinate synthase; *dapB*: dihydrodipicolinate reductase; *ddh*: diaminopimelate dehydrogenase; *fbp*: fructose-1, 6-bisphosphatase; *hom*: homoserine dehydrogenase; *icd*: isocitrate dehydrogenase; *lysA*: diaminopimelate decarboxylase; *lysC*: aspartokinase; *pck*: phosphoenolpyruvate carboxykinase; *pyc*: pyruvate carboxylase; *tal*: transaldolase; *tkt*: transketolase; *zwf*: glucose 6-phosphate dehydrogenase. 26dap-m: meso-2,6-diaminoheptanedioate; 23dhdp: (2S,4S)-4-hydroxy-2,3,4,5-tetrahydrodipicolinate; accoa: acetyl-CoA; akg: α-oxoglutarate; aspsa: l-aspartate 4-semialdehyde; cit: citrate; e4p: erythrose-4-phosphate; f6p: fructose-6-phosphate; fdp: fructose 1,6-bisphosphate; g3p: glyceraldehyde phosphate; g6p: glucose-6-phosphate; Glc: glucose; icit: isocitrate; l-asp: l-aspartate; l-hom: l-homoserine; l-lys: l-lysine; oaa: oxaloacetate; pep: phosphoenolpyruvate; pyr: pyruvate; ru5p-D: ribulose-5-phosphate; s7p: sedoheptulose-7-phosphate; succ: succinate; thdp: 2,3,4,5-tetrahydrodipicolinate; xu5p-D: xylulose-5-phosphate

To investigate whether *i*CW773 is an efficient tool to design strategies for the metabolic engineering of *C. glutamicum*, the ranges of flux variability in the network for the WT and l-lysine overproducing strains were calculated using OptForce_MUST_ (Additional file 6). As shown in Fig. 3, the gap between the flux ranges of the WT and overproducing network quantified the degree of required reaction flux modification. In the simulations of *i*CW773, the upregulation of aspartokinase (encoded by *lysC*), dihydrodipicolinate synthase (encoded by *dapA*), dihydrodipicolinate reductase (encoded by *dapB*), diaminopimelate dehydrogenase (encoded by *ddh*), and diaminopimelate decarboxylase (encoded by *lysA*) in the l-lysine synthesis pathway were consistent with previous reports [8, 44, 45]. Downregulation of homoserine dehydrogenase (encoded by *hom*) decreased the flux toward the competitive pathway [8, 44, 46]. Moreover, deletion of phosphoenolpyruvate carboxykinase (encoded by *pck*) and upregulation of pyruvate carboxylase (encoded by *pyc*) directed the flux toward oxaloacetate formation at the pyruvate node for l-lysine synthesis [8, 47]. Downregulation of aconitase (encoded by *acn*) and isocitrate dehydrogenase (encoded by *icd*) further minimized carbon loss from pyruvate [48]. In addition, upregulation of the entire transketolase operon and fructose-1,6-bisphosphatase (encoded by *fbp*) enhances the NADPH supply via PPP to increase l-lysine production [8, 49]. In contrast, Model_Cg_ 2 failed to predict the deletion of *pck*, the downregulation of *hom* and the upregulation of *lysC*, *ddh*, *dapB*, and *fbp*. Therefore, the predictions by *i*CW773 were highly consistent with experimental results. For l-valine and l-serine overproduction, the modification targets predicted by *i*CW773 were identical to the reported experimental results (Additional file 1: Table S2).

### Validation of *i*CW773 simulation for non-native 1,2-propanediol and isobutanol synthesis

**Fig. 4:**
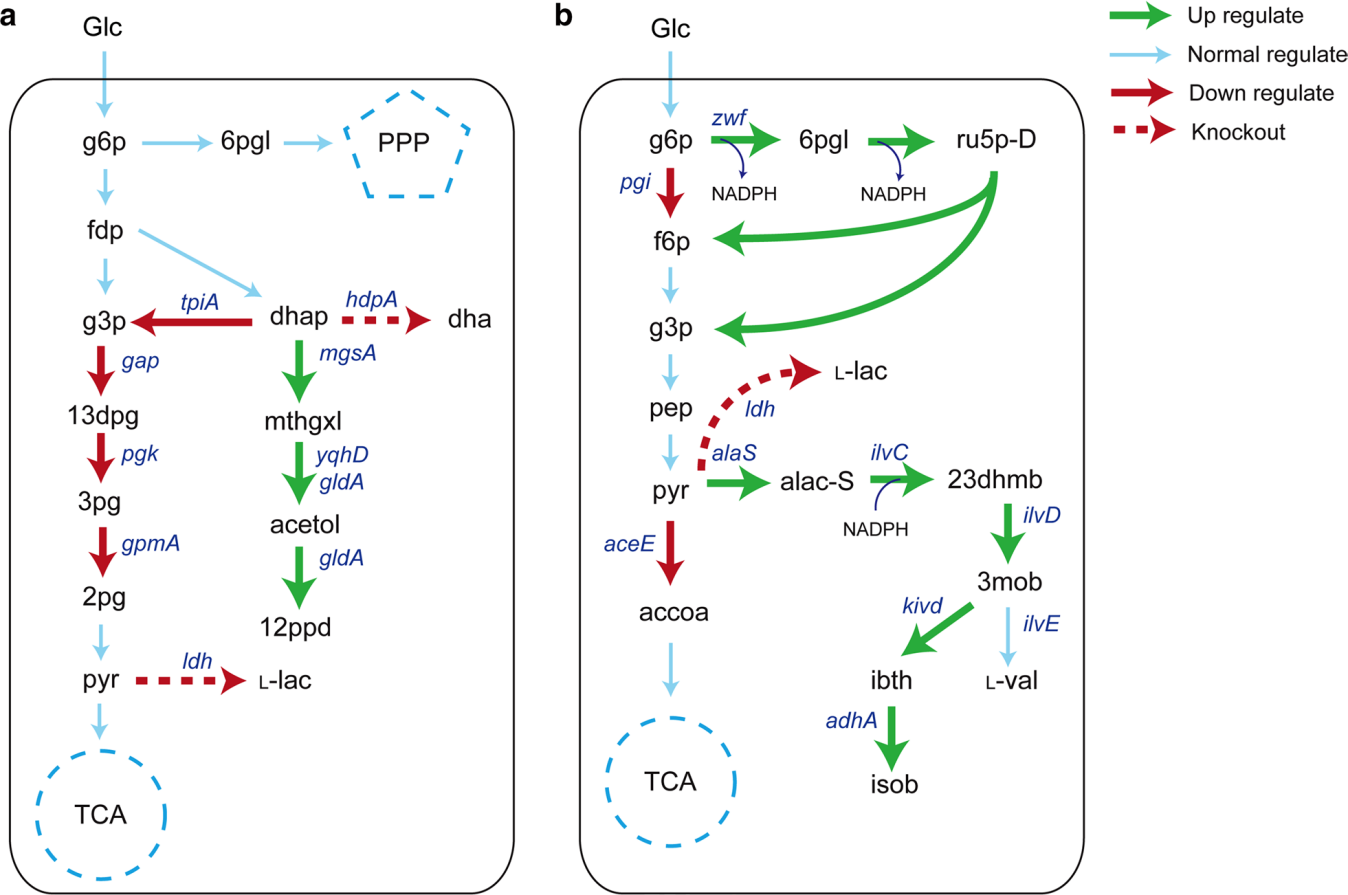
Application for overproduction of the non-natural products 1,2-propanediol (**a**) and isobutanol (**b**) using OptForce_MUST_. Genes encoding relevant enzymes are depicted with *italics* next to the *arrows*: *aceE*: pyruvate dehydrogenase complex; *adhA*: alcohol dehydrogenase; *alaS*: acetolactate synthase; *gap*: glyceraldehyde-3-phosphate dehydrogenase; *gldA*: glycerol dehydrogenase; *gpmA*: phosphoglycerate mutase; *hdpA*: dihydroxyacetone phosphate phosphatase; *ilvC*: acetohydroxyacid isomeroreductase; *ilvD*: dihydroxyacid dehydratase; *ilvE*: valine transaminase; *kivD*: keto acid decarboxylase; *ldh*: lactate dehydrogenase; *mgsA*: methylglyoxal synthase; *pgi*: glucose-6-phosphate isomerase; *pgk*: phosphoglycerate kinase; *tpiA*: triose-phosphate isomerase; *yqhD*: aldehyde reductase. For other genes, see Fig. 3. 12ppd: 1,2-propanediol; 13dpg: 3-phospho-d-glyceroyl phosphate; 23dhmp: 2,3-dihydroxy-3-methylbutanoate; 2pg: d-glycerate 2-phosphate; 3mob: 3-methyl-2-oxobutanoate; 3pg: 3-phospho-d-glycerate; 6pgl: 6-phospho-d-glucono-1,5-lactone; acetol: acetol; alas-S: 2-acetolactate; dha: dihydroxyacetone; dhap: dihydroxyacetone phosphate; ibth: isobutyraldehyde; isob: isobutanol; l-lac: l-lactate; mthgxl: methylglyoxal; l-val: l-valine. For other abbreviations, see Fig. 3

*Corynebacterium glutamicum* has previously been engineered for non-native 1,2-propanediol and isobutanol production [50, 51]. Therefore, we performed a comparison between *i*CW773-simulated and experimentally performed genetic modification strategies with respect to 1,2-propanediol and isobutanol overproduction [50, 51]. As previously reported, a three-reaction synthesis route by the heterologous genes *mgsA, gldA*, and *yqhD*, which encode methylglyoxal synthase, glycerol dehydrogenase, and alcohol dehydrogenase from *E. coli*, was added to *i*CW773 to synthesize 1,2-propanediol from dihydroxyacetone phosphate (dhap) [50]. Then, the ranges of flux variability in the network for the WT and 1,2-propanediol-overproducing strains were calculated by OptForce_MUST_ (Additional file 7). As shown in Fig. 4a, upregulation of *mgsA, gldA*, and *yqhD* directed the flux toward 1,2-propanediol synthesis, which was consistent with the finding that the heterologous expression of these three genes in the WT strain led to a product yield of 0.13 mol/mol (1,2-propanediol/glucose) [50]. Moreover, model simulation showed that the deletion of *hdpA* encoding dihydroxyacetone phosphate phosphatase could increase the precursor (dhap) supply by preventing glycerol formation from dhap, which was supported by the experimental results that 1,2-propanediol production increased by 90.08% in strain WT∆*hdpA* (pEKEx3-*mgsA*-*gldA*-*yqhD*) [50]. The OptForce_MUST_ analysis indicated that knockout of *ldh* encoding lactate dehydrogenase could further decrease the formation of byproducts and increase 1,2-propanediol production. This prediction was validated by the experimental finding that *ldh* deletion increased 1,2-propanediol by 37.75% [50]. In addition, downregulation of *tpiA*, *gap*, *pgk*, and *gpmA* would be novel targets for 1,2-propanediol overproduction.

As reported previously, the isobutanol pathway in *i*CW773 was constructed by *kivd*-encoding keto acid decarboxylase from *Lactococcus lactis* and native *adhA*-encoding alcohol dehydrogenase catalyzing 3-methyl-2-oxobutanoate to isobutanol [52]. The predicted knockout, upregulation, and downregulation of target genes by OptForce_MUST_ are shown in Fig. 4b (Additional file 7). The simulation was consistent with the experimental results that the upregulation of four genes (*alsS*, *ilvCD*, *kivd*, and *adhA*) could endow *C. glutamicum* with isobutanol synthesis capability [52]. The OptForce_MUST_ analysis indicated that the decrease in pyruvate consumption caused by inactivating *ldh*-encoding lactate dehydrogenase and downregulating the *aceE*-encoding pyruvate dehydrogenase subunit would increase isobutanol synthesis. In vivo *ldh* deletion increased the isobutanol yield by 23%, while deletion of *aceE* led to pyruvate overflow to lactate formation rather than increased isobutanol synthesis [52]. It was reported that *aceE* deletion caused *C. glutamicum* to be unable to grow with glucose unless supplemented with acetate, indicating that knockout of *aceE* has a negative effect on cell growth and that knockdown of *aceE*, as suggested by the *i*CW773 simulation, might be a better alternative [53]. In addition, the *i*CW773-simulated strategy to improve isobutanol synthesis included an increase in the intracellular NADPH pool by downregulation of *pgi* in combination with upregulation of *zwf*. This combined regulation mode for *pgi* and *zwf* had been demonstrated to increase intracellular NADPH levels by redirecting carbon flux toward PPP [9]. In contrast, deletion of *pgi* together with *aceE* severely inhibited growth and eliminated isobutanol production [52]. Taken together, these data indicate that downregulation of *aceE* and *pgi* together with upregulation of *zwf* would contribute to isobutanol synthesis and simultaneously maintain growth. In summary, the *i*CW773-designed strategy could provide insights into future work for the optimization of 1,2-propanediol and isobutanol production in *C. glutamicum*.

### In silico design and wet-lab construction of an l-proline-overproducing strain

**Fig. 5:**
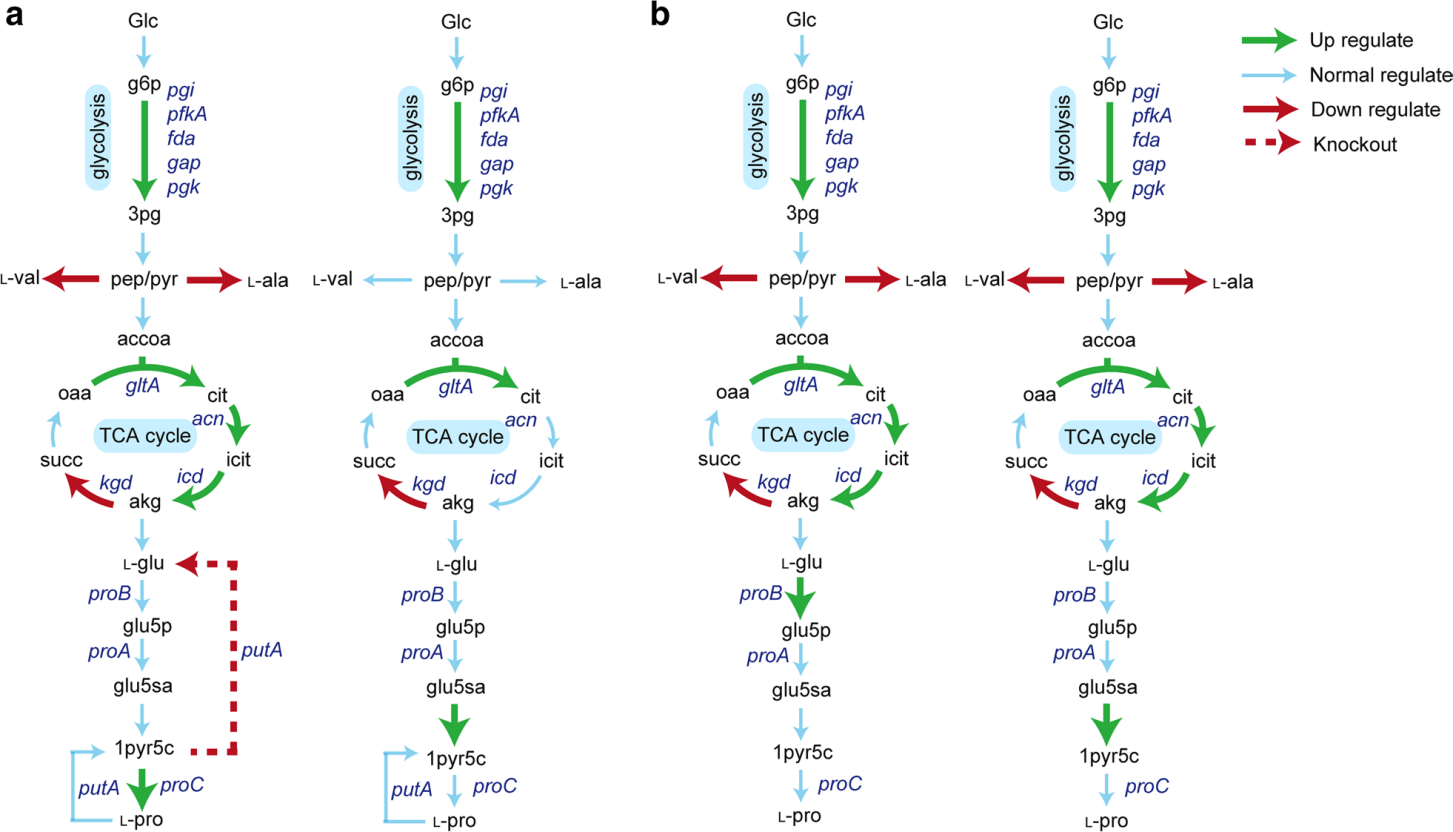
Metabolic interventions predicted using OptForce_MUST_ for l-proline overproduction in wild-type *C. glutamicum* ATCC13032 (**a**) predicted by *i*CW773 (*left*) and Model_Cg_ 2 (*right*) and in the mutant strain *C. glutamicum* ATCC13032 Δ*putA* (**b**) predicted by *i*CW773 (*left*) and Model_Cg_ 2 (*right*). Genes encoding relevant enzymes are depicted in *italics* next to the *arrows*: *fda*: fructose-bisphosphate aldolase; *gltA*: citrate synthase; *kgd*: α-oxoglutarate dehydrogenase; *pfkA*: phosphofructokinase; *proA*: glutamate-5-semialdehyde dehydrogenase; *proB*: γ-glutamyl kinase; *proC*: pyrroline-5-carboxylate reductase; *putA*: proline dehydrogenase and Δ1-pyrroline-5-carboxylate dehydrogenase. For other genes, see Figs. 3 and 4. 1pyr5c: Δ1-pyrroline-5-carboxylate; glu5p: glutamate-5-phosphate; glu5sa: glutamate-5-semialdehyde; l-ala: l-alanine; l-glu: l-glutamate; l-pro: l-proline. For other abbreviations, see Figs. 3 and 4

Despite the strong flux toward l-glutamate formation in *C. glutamicum*, l-proline converted from l-glutamate under three sequential catalytic reactions by γ-glutamyl kinase (*proB*), glutamate-5-semialdehyde dehydrogenase (*proA*), and pyrroline-5-carboxylate reductase (*proC*) has not been efficiently synthesized by the engineered strain derived from *C. glutamicum* ATCC13032 [54–56]. The heterologous expression of ornithine cyclodeaminase merely made an l-ornithine-producing strain overproduce l-proline by six enzymatic reactions from l-glutamate, which was not an economically feasible means of carbon utilization in l-proline synthesis [56, 57]. Thus, *i*CW773 was applied to identify metabolic interventions that led to the overproduction of l-proline via OptForce_MUST_ (Additional file 8). As shown in Fig. 5a, the upregulation of five reactions in the glycolytic pathway and citrate synthase (CS, encoded by *gltA*) and downregulation of α-oxoglutarate dehydrogenase (AKGDH, encoded by *kgd*) predicted by *i*CW773 were consistent with those predicted by Model_Cg_ 2. The differences between the *i*CW773 and Model_Cg_ 2 simulations were the downregulation of reactions from pyruvate to l-valine and l-alanine and upregulation of aconitase [ACONTa(b), encoded by *acn*] and isocitrate dehydrogenase (ICDHyr, encoded by *icd*), which converts citrate to α-oxoglutarate. The largest difference between the two models was the knockout of the *putA* gene to block the conversion of Δ1-pyrroline-5-carboxylate to glutamate. PutA is a bifunctional enzyme with both proline dehydrogenase and Δ1-pyrroline-5-carboxylate dehydrogenase activities, which catalyzes the two-step oxidation of l-proline to glutamate in *E. coli* [58]. In *i*CW773, the reaction from l-glutamate to Δ1-pyrroline-5-carboxylate formed a cycle in the presence of PutA, which made it inefficient to convert Δ1-pyrroline-5-carboxylate to l-proline. Blocking this conversion and upregulation of Δ1-pyrroline-5-carboxylate reductase (encoded by *proC*), the flux could be efficiently directed toward l-proline synthesis. Then, the PutA reactions were deleted in both models to predict the metabolic profile of overproducing l-proline (Additional file 9). As shown in Fig. 5b, one difference between the *i*CW773 and Model_Cg_ 2 simulations was the upregulation of ProB to direct the metabolic flux from l-glutamate toward l-proline synthesis. However, the reaction from glutamate-5-semialdehyde to Δ1-pyrroline-5-carboxylate, which differed in the Model_Cg_ 2 simulation, was impossible to control by genetic modification because this reaction occurred spontaneously without enzymatic catalysis.

**Fig. 6:**
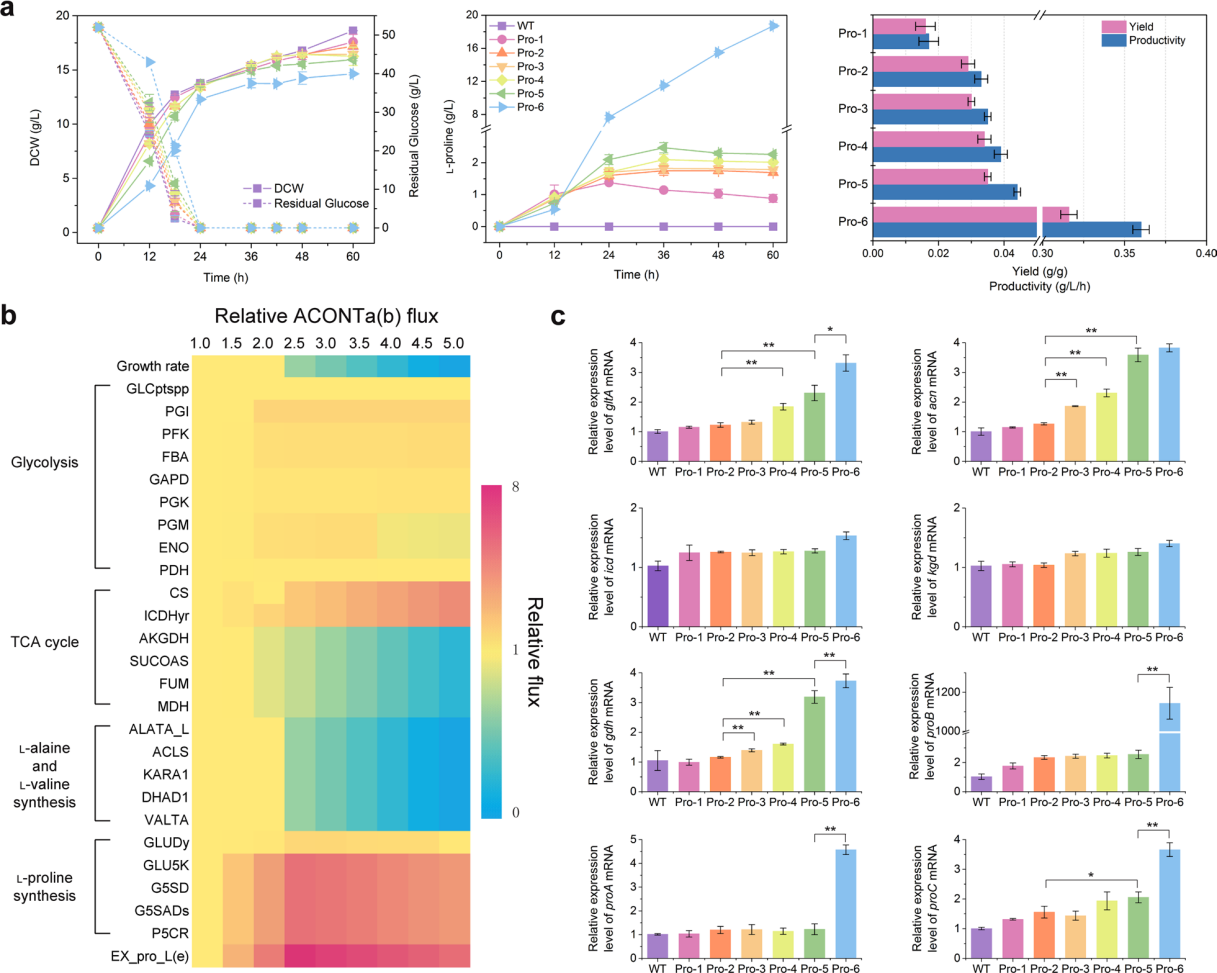
Analysis of l-proline-overproducing strains. **a** Flask cultivation of different *C. glutamicum* strains. **b** Simulation of the impact of increased ACONTa(b) flux on cellular metabolism for robustness analysis was investigated with increasing relative ACONTa(b) fluxes (between 1.0 and 5.0). *X-axis* relative ACONTa(b) flux (the ratio of the test ACONTa(b) flux to the native ACONTa(b) flux). Relative ACONTa(b) flux = 1.0 served as the control (or native GPD flux). *Y-axis* relative flux of the pathways (the ratio of the test flux to the native flux). *Yellow* represents no change, *red* represents upregulation, and *blue* represents downregulation of the flux. Descriptions of abbreviated reaction names are provided in Additional file 3. **c** Relative transcript expression of related genes of the analyzed *C. glutamicum* strains in the exponential growth phase in glucose. Significant differences in the data were determined using Student’s *t* test (*P < 0.05, **P < 0.01). The data were derived from experiments performed at least three times, and the *error bars* represent the standard deviations

To validate the accuracy of the *i*CW773 prediction for l-proline overproduction, different targets between the *i*CW773 and Model_Cg_ 2 simulations were chosen for genetic modification. Because the enzymatic activity of ProB is strictly restricted to feedback inhibition by l-proline, no accumulation of l-proline will occur in WT [59]. Therefore, the feedback inhibition of ProB by l-proline was first released through the introduction of the nucleotide substitution G446A into the chromosomal *proB* gene [60]. The Pro-1 strain accumulated a small amount of l-proline compared with the WT (Table 1). Notably, the l-proline titer dropped after 36 h (Fig. 6a), indicating that l-proline may be converted to l-glutamate under PutA catalysis as simulated by *i*CW773. When *putA* was knocked out in Pro-1, the l-proline production of Pro-2 was 1.92-fold higher than that of Pro-1 (Table 1), which was consistent with a previous report that deletion of *putA* significantly increased the formation of trans-4-l-hydroxyproline derived from l-proline in recombinant *E. coli* BL21 [61]. In addition, the product yield of l-proline from glucose was improved 1.94-fold upon deleting *putA* (Fig. 6a).

**Table 1:** Comparison of fermentation parameters of different strains in shake flask cultivation

Strain	*μ*_max_ (1/h)	DCW (g/L)	l-Alanine (g/L)	l-Valine (g/L)	Citrate (g/L)	α-Oxoglutarate (g/L)	Succinate (g/L)	l-Proline (g/L)	Yield_pro_ (mol/mol)
WT	0.27 ± 0.01	18.62 ± 0.02	4.98 ± 0.07	1.49 ± 0.24	6.88 ± 0.37	1.05 ± 0.14	8.01 ± 0.62	–	–
Pro-1 (WTproB*)	0.26 ± 0.00	17.60 ± 0.51	4.85 ± 0.07	1.37 ± 0.27	7.02 ± 0.56	1.08 ± 0.09	7.18 ± 0.80	0.88 ± 0.12	0.03 ± 0.00
Pro-2 (WTproB*∆*putA*)	0.25 ± 0.00	17.20 ± 0.46	4.72 ± 0.07	1.36 ± 0.28	7.54 ± 0.23	1.47 ± 0.20	7.44 ± 0.82	1.69 ± 0.10	0.05 ± 0.00
Pro-3 (WTproB*P_*pck*_::P_*acn*_ acn*∆*putA*)	0.26 ± 0.00	16.45 ± 0.13	4.54 ± 0.11	1.36 ± 0.37	5.48 ± 0.32	1.82 ± 0.29	7.25 ± 0.91	1.79 ± 0.02	0.05 ± 0.00
Pro-4 (WTproB*P_*glyA*_::P_*acn*_ acn*∆*putA*)	0.25 ± 0.00	16.27 ± 0.12	4.42 ± 0.04	1.30 ± 0.50	4.98 ± 0.16	2.05 ± 0.32	7.33 ± 0.86	2.01 ± 0.07	0.06 ± 0.00
Pro-5 (WTproB*P_*eftu*_::P_*acn*_ acn*∆*putA*)	0.23 ± 0.00	15.94 ± 0.10	3.92 ± 0.10	1.25 ± 0.10	2.01 ± 0.28	2.56 ± 0.09	8.19 ± 0.57	2.26 ± 0.03	0.07 ± 0.00
Pro-6 (WTproB*P_*eftu*_::P_*acn*_ acn*∆*putA*/pXMJ19-proB*)	0.20 ± 0.00	14.65 ± 0.07	2.12 ± 0.13	0.72 ± 0.05	1.63 ± 0.27	0.37 ± 0.01	4.47 ± 0.85	18.71 ± 0.21	0.56 ± 0.00

Upregulation of *gltA*, *acn*, and *icd* expression was simulated by *i*CW773 to drive more carbon flux to α-oxoglutarate formation for l-proline synthesis. It has been demonstrated that CS flux in vivo is not strictly controlled by the amount of *gltA* expression [62], while the transcription of the *acn* gene is subjected to complex regulations mediated by the AcnR, RipA, and GlxR repressors [63, 64]. Thus, aconitase-catalyzed reactions should be a major control point in the TCA cycle during growth on glucose [64, 65]. The effects of increased ACONTa(b) flux on cell metabolism and l-proline synthesis were simulated using FBA. The fluxes toward CS and ICDHyr were enhanced with the increase in the relative ACONTa(b) flux, and maximum extracellular l-proline production was achieved when the relative ACONTa(b) flux was improved by 2.5-fold compared with WT (Fig. 6b). Conversely, the fluxes toward l-valine and l-alanine synthesis decreased. After the native promoter of the *acn* gene was replaced with three promoters of varying strength (P_*glyA*_, P_*pck*_, and P_*eftu*_) and the start codon was changed from TTG to ATG in Pro-2, the mRNA levels of the *acn* gene increased by 1.47-, 1.82-, and 2.83-fold in the Pro-3, Pro-4 and Pro-5 strains, respectively (Fig. 6c). With upregulation of the *acn* gene, the mRNA levels of *gltA* in Pro-4 and Pro-5 increased by 50.82 and 88.52%, respectively, consistent with a previous observation that the expression of *gltA* and *acn* were simultaneously increased and might be controlled by a similar mechanism [65]. However, no significant changes were observed in the mRNA level of *icd* or *kgd* (Fig. 6c). When the mRNA levels of *acn* were increased, Pro-5 produced 2.26 ± 0.03 g/L l-proline with a 33.73% increase over Pro-2. Correspondingly, the byproducts of l-alanine and l-valine decreased by 16.95 and 8.09%. The extracellular metabolites exhibited a 73.34% decrease in citrate and a 1.37-fold increase in α-oxoglutarate.

Finally, to validate the positive correlation between GLU5K flux and l-proline yields (Additional file 4: Figure S2), the mutant *proB* (G446A) under the control of P_*tac*_ on a plasmid was induced to overexpress the feedback-resistant γ-glutamyl kinase. When the mRNA level of proB* was significantly increased, the mRNA levels of the *proA*, *proC*, and *gdh* genes were simultaneously increased (Fig. 6c). The resultant Pro-6 strain showed an 8.27-fold increase in l-proline production (18.71 ± 0.21 g/L), with 8.18- and 9.03-fold increases in yield (0.36 g/g) and productivity (0.32 g/L/h), respectively, compared with Pro-5 (Fig. 6a). In addition, there were 85.55 and 45.42% decreases in extracellular α-oxoglutarate and succinate, respectively.

### The performance of Pro-6 in fed-batch fermentation

**Fig. 7:**
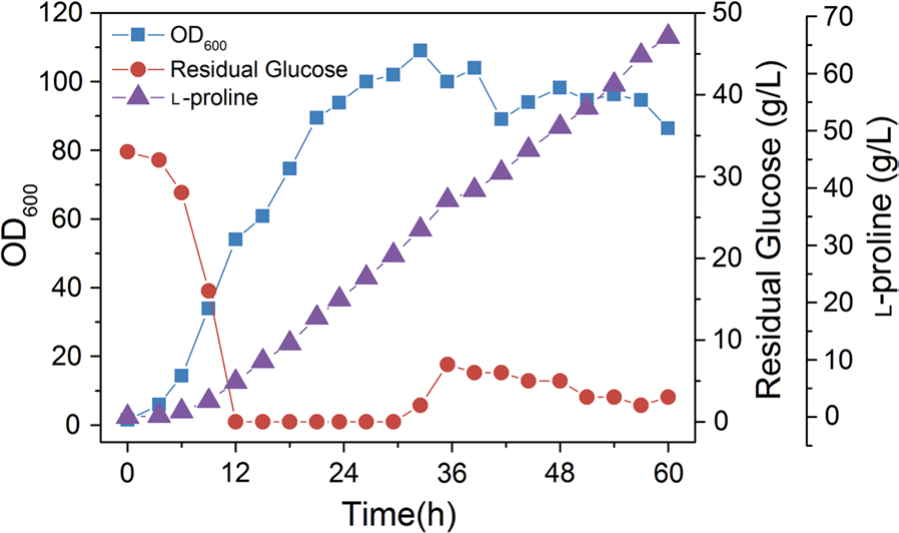
Fed-batch culture of Pro-6 in a 7.5-L bioreactor

The production performance of the final Pro-6 strain was investigated in fed-batch fermentation. As shown in Fig. 7, the strain grew continuously from 0 to 32 h and reached a cell concentration of OD_600_ = 109.02 at 32 h. The assimilated glucose was efficiently channeled to the l-proline biosynthetic pathway; l-proline production began at an early stage and continuously increased throughout the fermentation period. A major and constant increase in l-proline production was achieved during the feeding phase, which was initiated after the initial sugar (40 g/L) in the batch medium was consumed. The maximal specific growth rate of strain Pro-6 was 0.40 h^−1^, and the maximal specific glucose consumption rate was 5.46 mmol/gDCW/h. The maximal l-proline titer reached 66.43 g/L at 60 h with a yield of 0.26 g/g glucose (0.41 mol/mol glucose) and a productivity of 1.11 g/L/h. Consequently, the genetically engineered Pro-6 strain in this study was demonstrated to be an efficient l-proline-producing strain without any nutrition-auxotroph and with the highest titer and productivity reported on minimal medium with glucose as the sole carbon source (Table 2).

**Table 2:** Overview of l-proline-producing strains

Strain	Breeding	Medium	Titer (g/L)	Yield (g/g)	Productivity (g/L/h)	References
Pro-6	Metabolic engineering	Minimal medium	18.71 ± 0.21	0.36 ± 0.01	0.32 ± 0.01	This work
			66.43^b^	0.26^b^	1.11^b^	
*Corynebacterium glutamicum*	Metabolic engineering	Minimal medium		0.25–0.30^a^	0.25–0.30^a^	[57]
*Corynebacterium glutamicum*	Metabolic engineering	Minimal medium	12.70	0.36	0.42	[56]
*Corynebacterium acetoacidophilum*	l-Histidine auxotroph; mutation	YPD medium	42^b^		1.66^b^	[55]
*Serratia marcescens* SP511	Mutation	Complete medium	100^b^		1.04^ab^	[54]

^a^Estimated from reference

^b^Achieved in fed-batch fermentation

## Conclusions

In this study, we reconstructed a genome-scale metabolic model of *C. glutamicum* ATCC13032 with an expanded size and scope compared with the previous Model_Cg_ 2. The *i*CW773-simulated results of growth rates, metabolic flux profiles, and modification targets for overproducing natural and non-natural products were much more consistent with experimental data than those of Model_Cg_ 2. Moreover, in silico analysis of l-proline overproduction and modifications of only three genes (*putA*, *acn*, and *proB*) cooperatively forced carbon flux toward l-proline, with the highest titer and productivity reported on minimal medium. This work revealed that the genome-scale metabolic model successfully guided the wet-lab experiments. This development will further boost *C. glutamicum*, one of the most promising and valuable workhorses, into a new era of industrial microbial biotechnology beyond the classical field.

## Methods

### Genome-scale metabolic model reconstruction

The reconstruction of the *C. glutamicum* ATCC13032 metabolic network was conducted based on the model of Shinfuku et al. [22]. A 96-step procedure for metabolic network reconstruction was recently published [66], and the appropriate steps were followed after new genes, reactions, and metabolites were added to generate *i*CW773. The KEGG (http://www.genome.jp/kegg/) and UniProt (http://www.uniprot.org/) databases were referenced to obtain reaction information and reaction-gene associations during the draft reconstruction stage, and *i*JO1366 was referenced to obtain the abbreviations of the same metabolites between *C. glutamicum* and *E. coli* [17]. The charge and mass of reactions in *i*CW773 were automatically balanced using the CheckMassChargeBalance command in Cobra Toolbox, and reversibility was assigned based on the Gibbs free energy by the eQuilibrator database (http://equilibrator2.milolab.webfactional.com/). Cobra Toolbox 2.05 with MatLab 2010b (MathWorks Inc.) was used for additional model testing [67], and the glpk solvers were used for the optimization procedures [68].

### Flux balance analysis

The growth rates and metabolic flux distribution of *C. glutamicum* ATCC13032 were calculated using FBA with commercially available glpk and Matlab software [16, 18]. To simulate growth rates and metabolic flux distributions, biomass production was set as the objective function. For the simulation of growth rates under different glucose and oxygen levels, glucose and oxygen uptake rates were set as the experimental values, as shown in Fig. 1a. For the simulation of growth rates on different carbon sources, the uptake rate of related carbon source was set as the experimental value, as shown in Fig. 1b. Small molecular external metabolites such as CO_2_, H_2_O, SO_3_, NH_3_, and PO_4_ were allowed to be freely transported across the cell membrane.

### OptForce_MUST_

The flux variability analysis was conducted to calculate each reaction’s flux span for both the wild-type and overproducing strains. A ^13^C flux and a relatively high secretion rate analyses were employed to constraint the wild-type and overproducing-type simulations, respectively [69]. The flux ranges for each reaction were compared, and some reactions of the overproducing strain need to be genetically modified through up/downregulation or knockout if no overlap between the flux range of the wild-type strain and that of the overproducing strain was found. A pool of modified reactions to construct the overproducing-type strain was produced.

To analyze the overproductions of the native and non-native metabolites, ^13^C flux data of the wild-type strain on glucose under aerobic condition were used as the constraints of the maximal range of flux variability [8, 31, 41]. For the non-native product, simulations of 1,2-propanediol and isobutanol production were performed after the heterogeneous reactions were added to *i*CW773, as described in Additional file 10. We imposed a minimum production yield of 98% of the theoretical maximum for l-lysine, l-valine, l-serine, l-proline, 1,2-propanediol, and isobutanol; the biomass flux was constrained to at least 1% of its theoretical maximum; and the l-lysine, l-valine, l-serine, l-proline, 1,2-propanediol, and isobutanol transport reactions were set as the target reactions, respectively. The upper and lower bounds of the ^13^C-tracer experimental fluxes are listed in Additional file 10 [8, 31, 41]. OptForce_MUST_ was used to identify the reactions/genes that required to be up/downregulated or knocked out to maximize the production of the targeted metabolites.

### Strains and plasmids

Wild-type *C. glutamicum* ATCC13032 (American Type and Culture Collection, Manassas, VA, USA) was used as the parental strain for strain engineering. The *E. coli* strain EC135 was used as the cloning host, and the pXMJ19 and pK18*mobsacB* plasmids were applied for gene overexpression with induction of isopropyl-β-d-thiogalactopyranoside (IPTG) and gene deletion, respectively [70]. All of the strains and plasmids used in this study are listed in Additional file 1: Table S3.

### Constructions of plasmids and strains

Total genomic DNA was extracted from *C. glutamicum* according to a previously described procedure [71]. To construct a *C. glutamicum* strain carrying a point mutation resulting in an amino acid substitution from glycine to aspartate at position 149 of the ProB polypeptide, the *proB* gene was first deleted in the WT strain. The upstream and downstream homologous fragments of *proB* were amplified using PCR with primers P1/P2 and P3/P4. The amplified DNA fragments were spliced using overlap extension PCR and were ligated into the suicide vector pK18*mobsacB* [72]. The plasmids were verified by DNA sequencing and then transformed into the WT strain through electroporation to generate the WTΔ*proB* strain, which was further verified by sequencing. In a second step, the mutation was introduced into the *proB* gene by PCR amplification with the primers P1/P7 and P8/P4. The amplified DNA fragments were spliced using overlap extension PCR and ligated into the suicide vector pK18*mobsacB*. The obtained plasmid was transformed into the WTΔ*proB* strain to generate the Pro-1 (WTproB*) strain [60]. For deletion of the *putA* gene, previously isolated WT genomic DNA was amplified using PCR with primers P9/P10 and P11/P12. The amplified DNA fragments were spliced using overlap extension PCR and ligated into the suicide vector pK18*mobsacB*. The plasmids were verified by DNA sequencing and then transformed into the Pro-1 (WTproB*) strain through electroporation to generate the Pro-2 (WTproB*∆*putA*) strain, which was further verified by sequencing.

To replace the native promoter of the *acn* gene with the promoter of *pck*, the RBS sequence with ‘AAAGGAGGA,’ and the start codon with ATG, three fragments corresponding to the upstream region of *acn,* the introduced modulation element including the *pck* promoter and the RBS as well as the *acn* gene were amplified with primers P15/P16, P17/P18, and P19/P20, respectively. These fragments were spliced using overlap extension PCR and ligated into the suicide vector pK18*mobsacB*. The plasmids were verified by DNA sequencing and then transformed into the Pro-2 (WTproB*∆*putA*) strain through electroporation to generate the Pro-3 (WTproB*P_*pck*_::P_*acn*_ acn*∆*putA*) strain, which was further verified by sequencing. Similarly, the promotor of the *acn* gene was altered in Pro-2 (WT*proB* ^G446A^∆*putA*), which generated Pro-4 (WTproB*P_*glyA*_::P_*acn*_ acn*∆*putA*) and Pro-5 (WTproB*P_*eftu*_::P_*acn*_ acn*∆*putA*).

The *C. glutamicum* and *E. coli* shuttle vector pXMJ19 was used for the overexpression of the proB* gene [73]. For amplification of the proB* gene, genomic DNA isolated from Pro-1 (WTproB*) was amplified using PCR with primers P31/P32. Then, the amplified DNA fragment was ligated into the shuttle vector pXMJ19. The plasmids were verified by DNA sequencing and then transformed into the Pro-5 (WTproB*P_*eftu*_::P_*acn*_ acn*∆*putA*) strain through electroporation to generate the Pro-6 (WTproB*P_*eftu*_::P_*acn*_ acn*∆*putA*/pXMJ19-proB*) strain. When needed, 0.1 mM IPTG was added to the culture medium to induce target gene overexpression. The primers used in this study are provided in Additional file 1: Table S4.

### Media and cultivation

The *E. coli* strains were aerobically grown at 37 °C in Luria–Bertani medium [74]. The *C. glutamicum* strains were cultivated in brain heart infusion (BHI) medium (37 g/L brain heart infusion with 91 g/L sorbitol) at 30 °C for genetic disruption and complementation [75]. As a minimal medium, CGXII medium was used with 40 g/L glucose [76]. When necessary, antibiotics were added at the following concentrations: 50 μg/mL kanamycin or 20 μg/mL chloramphenicol for *E. coli* and 25 μg/mL kanamycin or 10 μg/mL chloramphenicol for *C. glutamicum*.

In the shake-flask growth experiment, *C. glutamicum* strains were precultured in the CGIII seed medium at 30 °C and 200 rpm until the OD_600_ reached 12. One milliliter of seed culture was inoculated in a 500-mL baffled shake flask with 30 mL of CGXII minimal medium. The cells were cultured in triplicate at 30 °C and shaken at 220 rpm. The pH was maintained at 7.0–7.2 via ammonia supplementation.

The fed-batch fermentation was performed in a 7.5-L bioreactor (BioFlo^®^/CelliGen^®^115, New Brunswick, USA) with a working volume of 2 L of CGX medium containing 40 g/L glucose. After 3 h of growth, 0.1 mmol/L IPTG was added for the induction of P_*tac*_. The concentration of glucose over all of the fed-batch cultures was maintained within the range of 5 ± 5 g/L by supplying 800 g/L of glucose reservoir. The glucose reservoir was fed into the fermenter at the rate of 0.2–0.5 mL/min according to the residual glucose concentration. The temperature was maintained at 32 °C using cold water circulation. The pH was maintained at 6.9 via the automated addition of ammonia and 100 mmol/L H_3_PO_4_. Dissolved oxygen was determined using a pO_2_ electrode and maintained above 30% saturation via variation of the stir speed.

### Analytical methods

The glucose concentration was measured using an SBA-40D biosensor analyzer (Institute of Biology of Shandong Province Academy of Sciences, Shandong, China). The cell concentration was determined by measuring the absorbance at 600 nm (OD_600_) using a spectrophotometer (V-1100D; Mapada Instruments, Shanghai, China). The dry cell weight (DCW) per liter was calculated using an experimentally determined formula: DCW (g/L) = 0.27 × OD_600_ [77]. The amino acids in the culture supernatant were determined using high-performance liquid chromatography with a Zorbax Eclipse XDB-C_18_ column (4.6 mm × 150 mm, 5 μm; Agilent) at 40 °C and 360 nm after derivatization with 2,4-dinitrofluorobenzene. Mobile phase A was 55% (v/v) acetonitrile, and mobile phase B consisted of 40 mmol/L KH_2_PO_4_ at pH 7.0–7.2. The flow rate of the mobile phase was 1 mL/min. The organic acids in the culture supernatant were determined using high-performance liquid chromatography with a SB-Aq column (4.6 × 250 mm; 5 μm; Agilent) at 40 °C and 210 nm. Mobile phase A was acetonitrile, and mobile phase B consisted of 20 mmol/L KH_2_PO_4_ at pH 2.3. The flow rate of the mobile phase was 1 mL/min.

### RNA preparation and quantitative RT-PCR

*Corynebacterium glutamicum* strains were grown to the exponential phase in CGXII minimal medium as described above. The cells were harvested and the total RNA was isolated using an RNAprep Pure Cell/Bacteria Kit (Tiangen, China). Reverse transcription of approximately 400 ng of RNA was performed with the primers listed in Additional file 1: Table S4 using a FastQuant RT Kit (Tiangen, China). Quantitative PCR was performed using GoTaq qPCR master mix (Promega, USA) in a 20-μL mixture with a LightCycler^®^ 96 Real-Time PCR System (Roche, Switzerland). The *C. glutamicum rpoB* gene was used as the reference gene to normalize the *gltA*, *acn*, *icd*, *kgd*, *gdh*, *proB*, *proA*, and *proC* mRNA levels [70]. Negative controls were used in each PCR run to exclude DNA and other contaminants. The qPCR products were verified via a melting curve analysis. Data collection and analyses were conducted using LightCycler^®^ 96 software (Roche, Switzerland) with the 2^−∆∆CT^ method [78].

## Additional files

**Additional file 1: Table S1.** Comparison of GEM attributes among various *C. glutamicum* models. **Table S2.** Comparison between the in silico prediction of the genes and proteins involved in the overproduction of l-valine and l-serine with the experimental data. **Table S3.** Strains and plasmids used in this study. **Table S4.** Primers used in this study.

**Additional file 2.** The lumped reactions of Model_Cg_ 2.

**Additional file 3.** The reactions and metabolites of *i*CW773.

**Additional file 4: Figure S1.** The central metabolic network of *C. glutamicum*. **Fig. S2.** Robustness analysis of GLU5K flux on l-proline production rate by Pro-2 and Pro-5.

**Additional file 5.** The flux distribution values for simulations on acetate, lactate and xylose using FBA by *i*CW773.

**Additional file 6.** Overproduction of l-lysine in WT using OptForce_MUST_ by *i*CW773 and Model_Cg_ 2.

**Additional file 7.** Overproduction of 1,2-propanediol and isobutanol in WT using OptForce_MUST_ by *i*CW773.

**Additional file 8.** Overproduction of l-proline in WT using OptForce_MUST_ by *i*CW773 and Model_Cg_ 2.

**Additional file 9.** Overproduction of l-proline in WTΔ*putA* using OptForce_MUST_ by *i*CW773 and Model_Cg_ 2.

**Additional file 10.** The upper and lower bounds of the ^13^C-tracer experimental fluxes used by OptForce_MUST_ and the added reactions used in this study.

## Abbreviations

Δ: deletion
ACONTa(b): aconitase
*C. glutamicum*: *Corynebacterium glutamicum*
CS: citrate synthase
DCW: dry cell weight
*E. coli*: *Escherichia coli*
FBA: flux balance analysis
FVA: flux variability analysis
GEM: genome-scale metabolic model
GLU5K: γ-glutamyl kinase
HPLC: high-performance liquid chromatography
IPTG: isopropylthio-β-d-galactopyranoside
NADPH and NADP: reduced and oxidized form of nicotinamide adenine dinucleotide phosphate, respectively
OD_600_: optical density at wavelength (*λ*) 600 nm
ORF: open reading frame
PPP: pentose phosphate pathway
PYRC: pyruvate carboxylase
TCA: tricarboxylic acid cycle
WT: wild-type
