# Supplementary material for: A new genome-scale metabolic model of Corynebacterium glutamicum and its application
Source: Biotechnol Biofuels. 2017 Jun 30;10:169. doi: 10.1186/s13068-017-0856-3 (PMC5493880; doi:10.1186/s13068-017-0856-3)
Supplement: Supplementary file 1 — Additional file 1: Table S1. Comparison of GEM attributes among various C. glutamicum models. Table S2. Comparison between the in silico prediction of the genes and proteins involved in the overproduction of l-valine and l-serine with the experimental data. Table S3. Strains and plasmids used in this study. Table S4. Primers used in this study. [file 13068_2017_856_MOESM1_ESM.docx]

**Tables**

**Table S1** Comparison of GEM attributes among various *C. glutamicum* models.

| Features | *C.glutamicum*  ATCC 13032 | | | *C.glutamicum*  S9114 |
| --- | --- | --- | --- | --- |
| **Genomic information** |  |  |  |  |
| Genome size (Mb) | 3.28 | 3.28 | 3.28 | 3.29 |
| No. of open reading frames (ORFs) | 3002 | 3002 | 3002 | 3098 |
| **Reconstructed metabolic model** | *i*CW773 | Model_Cg_ 1 | Model_Cg_ 2 | *i*JM658 |
| **Total reactions** | 1207 | 446 | 502 | 1065 |
| Internal reactions | 795 | 304 | 441 | 811 |
| Transport reactions | 252 | 55 | 29 | 165 |
| Exchange reactions | 160 | 87 | 32 | 89 |
| **Metabolites** | 951 | 411 | 423 | 984 |
| Unique metabolites | 746 | 341 | 396 | 847 |
| **No. of ORFs including in the model** | 773 | 247 | 277 | 658 |
| **ORF coverage (%)** | 25.75% | 8.23% | 9.23% | 21.80% |

**Table S2** Comparison between the *in silico* prediction of the genes and proteins involved in the overproduction of l-valine and l-serine with the experimental data.

| Amino  acids | Reaction | Experiment | *i*CW773 | WT LB^a^ | WT UB^a^ | OP LB^a^ | OP UB^a^ | CDS | Gene | Protein | Reference |
| --- | --- | --- | --- | --- | --- | --- | --- | --- | --- | --- | --- |
| l-valine | ACLS | upregulation | upregulation | 0.12 | 1.59 | 4.54 | 4.63 | *cg1435*  *cg1436* | *ilvBN* | acetolactate synthase | [[1](#_ENREF_1)] |
|  | KARA1 | upregulation | upregulation | 1.59 | 0.12 | 4.62 | 4.53 | *cg1437* | *ilvC* | ketol-acid reductoisomerase | [[1](#_ENREF_1)] |
|  | DHAD1 | upregulation | upregulation | 0.12 | 1.58 | 4.53 | 4.62 | *cg1432* | *ilvD* | dihydroxy-acid dehydratase | [[1](#_ENREF_1)] |
|  | THRD_L | knockout | downregulation | 0.03 | 0.08 | 0.00 | 0.00 | *cg2334* | *ilvA* | l-threonine deaminase | [[1](#_ENREF_1)] |
|  | MOHMT | knockout | knockout | 0.00 | 0.00 | 0.00 | 0.00 | *cg0149* | *panB* | 3-methyl-2-oxobutanoate  hydroxymethyl transferase | [[1](#_ENREF_1)]  [[2](#_ENREF_2)] |
|  | PANTS | knockout | knockout | 0.00 | 0.00 | 0.00 | 0.00 | *cg0148 cg2975* | *panC* | pantoate-beta-alanine ligase | [[1](#_ENREF_1)] |
|  | PDH | knockout | knockout | 0.00 | 0.00 | 0.00 | 0.00 | *cg2466* | *aceE* | pyruvate dehydrogenase | [[3](#_ENREF_3)] |
| l-serine | GHMT2r | knockout | knockout | 0.09 | 0.72 | 0.00 | 0.00 | *cg1133* | *glyA* | glycine hydroxymethyltransferase | [[4](#_ENREF_4), [5](#_ENREF_5)] |
|  | SERD_L | knockout | knockout | 0.01 | 0.63 | 0.00 | 0.00 | *cg1852* | *sdaA* | l-serine deaminase | [[4](#_ENREF_4), [5](#_ENREF_5)] |
|  | PGCD | upregulation | upregulation | 0.39 | 0.81 | 1.04 | 46.78 | *cg1451* | *serA* | phosphoglycerate dehydrogenase | [[4](#_ENREF_4), [5](#_ENREF_5)] |
|  | PGK | upregulation | upregulation | 7.20 | 7.37 | 8.05 | 9.32 | *cg1790* | *pgk* | phosphoglycerate kinase | [[4](#_ENREF_4)] |
|  | PSP_L | upregulation | upregulation | 0.39 | 0.81 | 1.04 | 46.78 | *cg2779* | *serB* | phosphoserine phosphatase | [[5](#_ENREF_5)] |
|  | PSERT | upregulation | upregulation | 0.39 | 0.81 | 1.04 | 46.78 | *cg0948* | *serC* | phosphoserine aminotransferase | [[5](#_ENREF_5)] |

^a^ WT LB: wild-type lower bound; WT UB: wild-type upper bound; OP LB: Overproduction-type lower bound; OP UB: Overproduction-type upper bound.

**Table S3** Strains and plasmids used in this study.

| Strains or plasmids | Relevant characteristicsa ^a^ | Source or reference |
| --- | --- | --- |
| Strains |  |  |
| *E.coli* |  |  |
| EC135 | *E.coli* TOP10∆*dcm*::*FRT recA*+ ∆*dam*::*FRT*, genotype of R-M systems:  *mcrA*∆(*mrr*-*hsdRMS*-*mcrBC*) ∆*dcm*::*FRT* ∆*dam*::*FRT* | [[6](#_ENREF_6)] |
| *C. glutamicum* |  |  |
| WT | ATCC13032, Wild-type strain | ATCC |
| Pro-1 | WT with mutation G446A into *proB* gene | This study |
| Pro-2 | WT*proB*^*^ with deletion in *putA* | This study |
| Pro-3 | WT*proB*^*^ ∆*putA* with replacement of the natural promoter of the *acn* gene by the promoter of *pck*, mutation T1A into *acn* gene and strong RBS sequence ‘AAAGGAGGA’ | This study |
| Pro-4 | WT*proB*^*^ ∆*putA* with replacement of the natural promoter of the *acn* gene by the promoter of *glyA*, mutation T1A into *acn* gene and strong RBS sequence ‘AAAGGAGGA’ | This study |
| Pro-5 | WT*proB*^*^ ∆*putA* with replacement of the natural promoter of the *acn* gene by the promoter of *eftu*, mutation T1A into *acn* gene and strong RBS sequence ‘AAAGGAGGA’ | This study |
| Pro-6 | WT*proB*^*^P*_eftu_*::P*_acn_acn*^*^∆*putA* with pXMJ19-*proB*^*^ | This study |
| Plasmids |  |  |
| pK18*mobsacB* | Kan^r^; shuttle vector for the construction of deletion mutants | [[7](#_ENREF_7)] |
| pK18*mobsacB* -∆*proB* | Kan^r^; pK18*mobsacB* with DNA fragment for the deletion of *proB* | This study |
| pK18*mobsacB* –*proB*^*^ | Kan^r^; pK18*mobsacB* with DNA fragment for the replacement of *proB*^G446A^ | This study |
| pK18*mobsacB* -∆*putA* | Kan^r^; pK18*mobsacB* with DNA fragment for the deletion of *putA* | This study |
| pK18*mobsacB*–P*_acn_*::P*_pck_*-RBS-*acn^*^* | Kan^r^; pK18*mobsacB* with DNA fragment for the replacement of the natural promoter of the *acn* gene by the promoter of *pck*, mutation T1A into *acn* gene and strong RBS sequence ‘AAAGGAGGA’ | This study |
| pK18*mobsacB*–P*_acn_*::P*_glyA_*-RBS-*acn^*^* | Kan^r^; pK18*mobsacB* with DNA fragment for the replacement of the natural promoter of the *acn* gene by the promoter of *glyA*, mutation T1A into *acn* gene and strong RBS sequence ‘AAAGGAGGA’ | This study |
| pK18*mobsacB*–P*_acn_*::P*_eftu_*-RBS-*acn^*^* | Kan^r^; pK18*mobsacB* with DNA fragment for the replacement of the natural promoter of the *acn* gene by the promoter of *eftu*, mutation T1A into *acn* gene and strong RBS sequence ‘AAAGGAGGA’ | This study |
| pXMJ19 | Cm^r^; Shuttle vector for expression of proteins (P_tac_, *lacI*^q^) | [[8](#_ENREF_8)] |
| pXMJ19- *proB*^*^ | Cm^r^; pXMJ19 derivative for expression of *proB*^G446A^ | This study |

^a^Abbreviations: Kan, Kanamycin; Cm, chloramphenicol.

**Table S4** Primer used in this study.

| Primer | Sequence^a^(5'-3') | Note |
| --- | --- | --- |
| P1 | CCGGAATTCCAAGTTGGGCATTGAGGACG *Eco*RI | pK18mobsacB-∆*proB* |
| P2 | CAGCAGGCCCGCGCTTCCGGATTCATGTCCGTAT | Up fragment (P1/P2) |
| P3 | GGACATGAATCCGGAAGCGCGGGCCTGCTGGTGGCGG | Down fragment (P3/P4) |
| P4 | CCCAAGCTTGGCCGCACGCTCCACG *Hin*dIII |  |
| P5 | ATCACCGCACTAAGGGGCAGTTCCA | Colony PCR (P5/P6) |
| P6 | GGACGACCAGAGTTATTAACCGCAA |  |
| P7 | GTCACCAAAATTCACATCGGTGGTTGCCACGGT | pK18*mobsacB* –*proB*^G446A^  Up fragment (P1/P7) |
| P8 | ACCGTGGCAACCACCGATGTGAATTTTGGTGAC | Down fragment (P8/P4) |
| P9 | CCCAAGCTTGGTCAATGTCGGTGATGATCCT *Hin*dIII | pK18*mobsacB* -∆*putA* |
| P10 | CCATGCGCAAAACGAGGTGGTTCTCCTTCAAGATCAG | Up fragment (P9/P10) |
| P11 | TGAAGGAGAACCACCTCGTTTTGCGCATG | Down fragment (P11/P12) |
| P12 | ACGCGTCGACACGGTCACGCCGTGCTCCA *Sal*I |  |
| P13 | CTAGGCCAATGGCTTGAGCTGCGGT | Colony PCR (P13/P14) |
| P14 | GCTCCCGCTCCGACTCGCCACCCTC |  |
| P15 | CCGGAATTCAAAATCTGATTCCTTTGCA *Eco*RI | pK18*mobsacB*–P*_acn_*::P*_pck_*-RBS-*acn^T1A^* |
| P16 | CTATTTTGGGGGTGGTTTCACTTCATTATCCTAACAGTA | Up fragment (P15/P16) |
| P17 | GTACTGTTAGGATAATGAAGTGAAACCACCCCCAAAATAG | P*_acn_*::P*_pck_* fragment (P17/P18) |
| P18 | TCACAGTGAGCTCCATTTCTATCCTCCTTTTACTTCTCCAGATTTTGTG |  |
| P19 | AAATCTGGAGAAGTAAAAGGAGGATAGAAATGGAGCTCACTGTGACTGA | Down fragment (P19/P20) |
| P20 | CCCAAGCTTTGGTGGTGTGGGAGTCG *Hin*dIII |  |
| P21 | ATCACACTAGTGGAGTAGCTCTTCATTATCCTAACAGTACA | pK18*mobsacB*–P*_acn_*::P*_glyA_*-RBS-*acn^T1A^*  Up fragment (P15/P21) |
| P22 | GTACTGTTAGGATAATGAAGAGCTACTCCACTAGTGTGAT | P*_acn_*::P*_glyA_* fragment (P22/P23) |
| P23 | AGTCACAGTGAGCTCCATTTCTATCCTCCTTTCAGGTCAGCTAACCTTT |  |
| P24 | AAAGGTTAGCTGACCTGAAAGGAGGATAGAAATGGAGCTCACTGTGACT | Down fragment (P24/P20) |
| P25 | TTCGCAGGGTAACGGCCACTTCATTATCCTAACAGTACAA | pK18*mobsacB*–P*_acn_*::P*_eftu_*-RBS-*acn^T1A^*  Up fragment (P15/P25) |
| P26 | GTACTGTTAGGATAATGAAGTGGCCGTTACCCTGCGA | P*_acn_*::P*_eftu_* fragment (P26/P27) |
| P27 | AGTCACAGTGAGCTCCATTTCTATCCTCCTTTTGTATGTCCTCCTG |  |
| P28 | ATACAAAAGGAGGATAGAAATGGAGCTCACTGTGACTGAA | Down fragment (P28/P20) |
| P29 | TAATCAGTGGTCCCAAGCAAATCAT | Colony PCR (P29/P30) |
| P30 | CGTCGATTGGGAACATCGCACAGGT |  |
| P31 | CCCAAGCTTAAAGGAGGACCGGAATGCGTGAG *Hin*dIII | pXMJ19- *proB*^G446A^(P31/P32) |
| P32 | GATTCTAGATTACGCGCGGCTGGCGTAGT *Xba*I |  |
| P33 | CGTACTGCTGAAGGCTCTT | *rpoB* for RT-PCR |
| P34 | TTTGCTACACCATCGGACT |  |
| P35 | ACCTACACCGACGACGCTGTTTCCG | *acn* for RT-PCR |
| P36 | GTTGTCAGCTTCGACGCCGCCTTCA |  |
| P37 | TTCGTATGATCGGTTCCGCACAGGC | *gltA* for RT-PCR |
| P38 | GTCGCCACCGTGGTTGCTCTTGATG |  |
| P39 | ACCGTTATCGAAGACTGCCGCAAGA | *icd* for RT-PCR |
| P40 | TGAACCACACCGTCTGCTTCGATGC |  |
| P41 | CCAAAGCCAACCCAGGCAGAGCAGA | *kgd* for RT-PCR |
| P42 | GCGGAGTCCATCAGTGGGATAAGTG |  |
| P43 | TCCGATTCCAGCGGTTGGGTTCATA | *gdh* for RT-PCR |
| P44 | GGTAGGTTGCGCCTTCAACTTCGTC |  |
| P45 | TTTATTTCCGAGGTTCGTGACGGCA | *proB* for RT-PCR |
| P46 | CGCAGAGGTCAACAGCACAGGCACG |  |
| P47 | CGCTCCAGGAAGCAGGAGTCACAAT | *proA* for RT-PCR |
| P48 | ACCGTCAACCACAGCGACAGCGATA |  |
| P49 | CCCCTGCATACCTGTTCCTTGTGAC | *proC* for RT-PCR |
| P50 | TTGCCGGTTTCCTTCATCATGGTTG |  |

^a^The restriction sites are underlined.

References

1. Radmacher E, Vaitsikova A, Burger U, Krumbach K, Sahm H, Eggeling L: Linking central metabolism with increased pathway flux: L-valine accumulation by *Corynebacterium glutamicum*. *Applied and Environmental Microbiology* 2002, 68:2246-2250.

2. Bartek T, Makus P, Klein B, Lang S, Oldiges M: Influence of L-isoleucine and pantothenate auxotrophy for L-valine formation in *Corynebacterium glutamicum* revisited by metabolome analyses. *Bioprocess and Biosystems Engineering* 2008, 31:217-225.

3. Blombach B, Schreiner ME, Holatko J, Bartek T, Oldiges M, Eikmanns BJ: (L)-Valine production with pyruvate dehydrogenase complex-deficient *Corynebacterium glutamicum*. *Applied and Environmental Microbiology* 2007, 73:2079-2084.

4. Lai SJ, Zhang Y, Liu SW, Liang Y, Shang XL, Chai X, Wen TY: Metabolic engineering and flux analysis of for *Corynebacterium glutamicum* for L-serine production. *Science China-Life Sciences* 2012, 55:283-290.

5. Peters-Wendisch P, Stolz M, Etterich H, Kennerknecht N, Sahm H, Eggeling L: Metabolic engineering of *Corynebacterium glutamicum* for L-serine production. *Applied and Environmental Microbiology* 2005, 71:7139-7144.

6. Zhang GQ, Wang WZ, Deng AH, Sun ZP, Zhang Y, Liang Y, Che YS, Wen TY: A Mimicking-of-DNA-Methylation-Patterns Pipeline for Overcoming the Restriction Barrier of Bacteria. *Plos Genetics* 2012, 8.

7. Schafer A, Tauch A, Jager W, Kalinowski J, Thierbach G, Puhler A: Small Mobilizable Multipurpose Cloning Vectors Derived from the *Escherichia-Coli* Plasmids Pk18 and Pk19 - Selection of Defined Deletions in the Chromosome of Corynebacterium-Glutamicum. *Gene* 1994, 145:69-73.

8. Jakoby M, Ngouoto-Nkili CE, Burkovski A: Construction and application of new *Corynebacterium glutamicum* vectors. *Biotechnology Techniques* 1999, 13:437-441.
