# Supplementary material for: A new genome-scale metabolic model of Corynebacterium glutamicum and its application
Source: Biotechnol Biofuels. 2017 Jun 30;10:169. doi: 10.1186/s13068-017-0856-3 (PMC5493880; doi:10.1186/s13068-017-0856-3)
Supplement: Supplementary file 4 — Additional file 4: Figure S1. The central metabolic network of C. glutamicum. Fig. S2. Robustness analysis of GLU5K flux on l-proline production rate by Pro-2 and Pro-5. [file 13068_2017_856_MOESM4_ESM.docx]

**Figures**


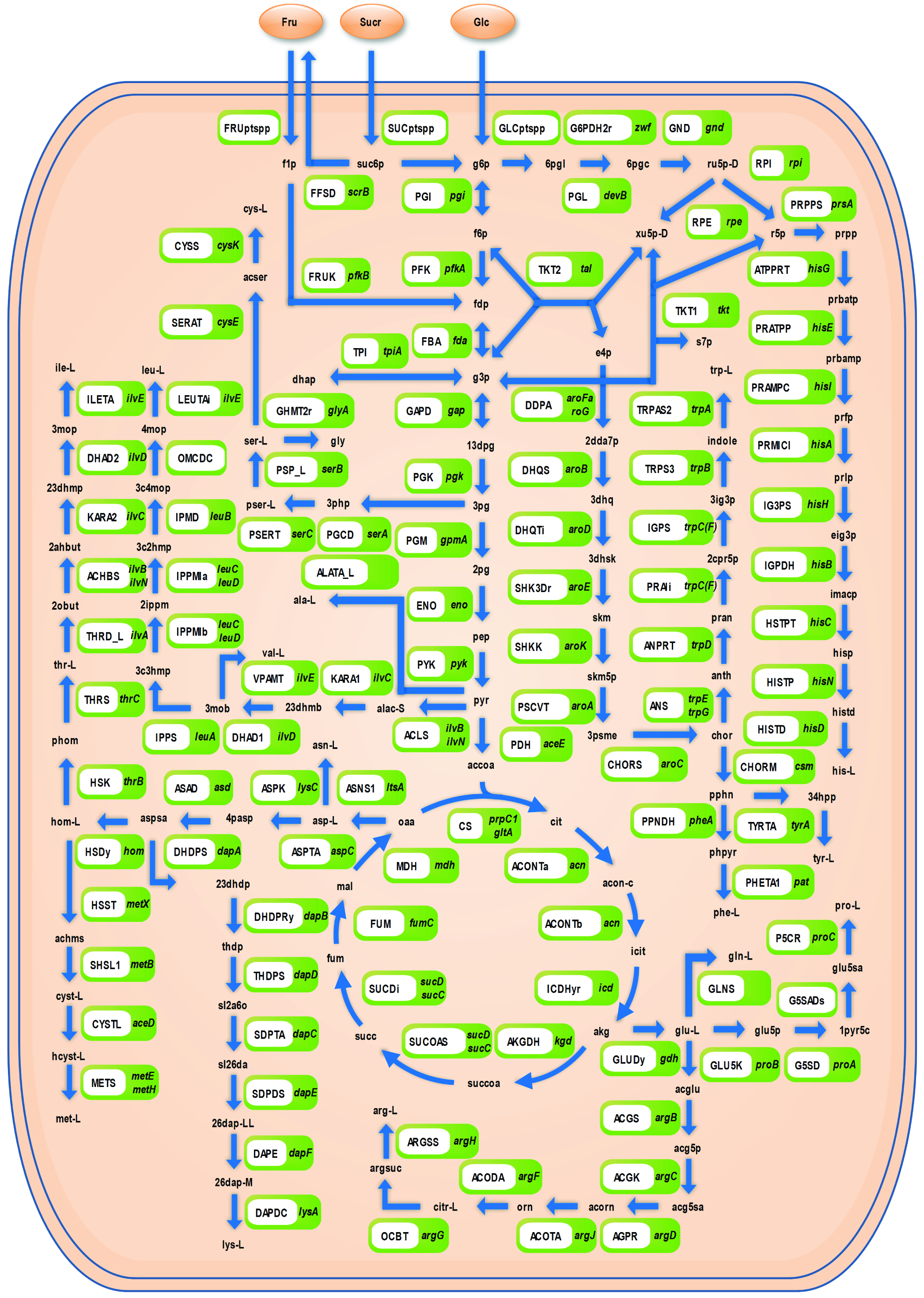


**Fig. S1** The central metabolic network of *C. glutamicum*.


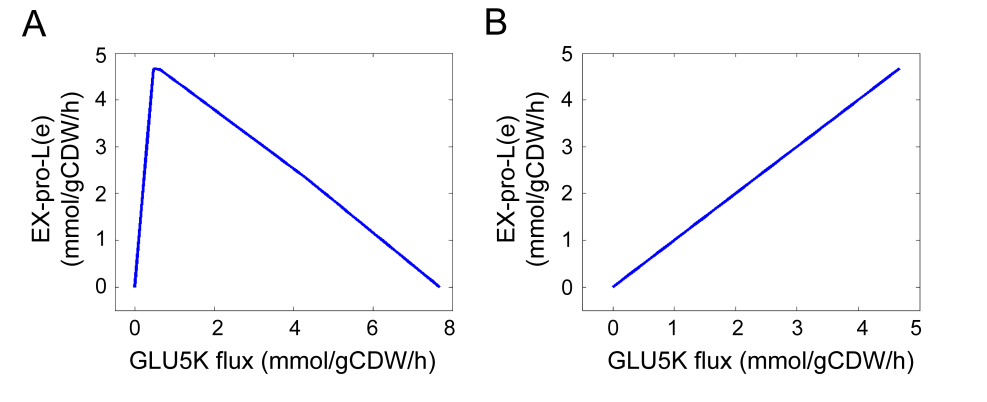


**Fig. S2** Robustness analysis of GLU5K flux on l-proline production rate by Pro-2 (**A**) and Pro-5 (**B**).
